# Characterization of primary mammary epithelial cells with loss of *BRCA1* at a single cell level

**DOI:** 10.1186/1753-6561-7-S2-P58

**Published:** 2013-04-04

**Authors:** Rebecca E Nakles, Sarah Millman, M Carla Cabrera, Peter Johnson, Susette C Mueller, Philipp S Hoppe, Timm Schroeder, Priscilla A Furth

**Affiliations:** 1 Department of Oncology, Georgetown University, Washington, DC, USA; 2 Department of Medicine, Georgetown University, Washington, DC, USA; 3 Lombardi Comprehensive Cancer Center, Georgetown University, Washington, DC, USA; 4 Helmholtz Zentrum München – German Research Center for Environmental Health, Ingolstaedter Landstraße 1, D-85764 Neuherberg, Germany; 5 Department of Nanobiomedical Science and WCU Research Center of Nanobiomedical Science, Dankook University, Chungnam 330-714, Korea

## Background

Loss of *BRCA1* has been linked to increased cell proliferation in human mammary epithelial cells. To determine if this phenotype is mirrored in the normal-appearing mammary epithelial cells from mouse models of BRCA1 deficiency, time-lapse imaging was performed on primary mammary epithelial cell (PMEC) cultures. Three distinct genetic models were tested to evaluate the role of p53 haploinsufficiency and p53 haploinsufficiency in the background of ERα over-expression to altered cell proliferation.

## Methods

PMEC cultures were generated from 10-12 month old wild-type, *Brca1f11/f11/MMTV−Cre*, *Brca1f11/f11/MMTV−Cre/p53*+*/-* and *Brca1f11/f11/MMTV−Cre/p53*+*/−/Tet-op-ER/MMTV-rtTA* mice. Live cell phase contrast time-lapse imaging performed for 5-7 days immediately after plating. Timm’s Tracking Tool software (http://www.helmholtz-muenchen.de/scd/service/scientific-services/software-downloads/index.html) was used to measure individual cell lifetimes.

## Results

Mean cell lifetimes in generations 1-4 were significantly shorter in PMEC cultures from *Brca1f11/f11/MMTV−Cre* and *Brca1f11/f11/MMTV−Cre/p53*+*/−* mice as compared to *Brca1f11/f11/MMTV−Cre/p53*+*/−/Tet-op-ER/MMTV-rtTA* and wild-type mice. A higher percentage of dividing cells were found in *Brca1f11/f11/MMTV−Cre*, *Brca1f11/f11/MMTV−Cre/p53*+*/−* and *Brca1f11/f11/MMTV−Cre/p53*+*/−/Tet-op-ER/MMTV-rtTA* mice as compared to wild-type mice. *Brca1f11/f11/MMTV−Cre/p53*+*/−/Tet-op-ER/MMTV-rtTA* mice showed the highest level of colony formation and lowest numbers of apoptotic cells. *Brca1f11/f11/MMTV−Cre/p53*+*/−* mice showed the lowest level of colony formation and highest number of apoptotic cells.

## Conclusions

Loss of Brca1 by itself was sufficient to decrease cell lifetime however this was modifiable by exposure to ERα overexpression. An inverse relationship between colony formation and numbers of apoptotic cells was found. In summary, genotype-specific differences in primary mammary epithelial cell behavior were revealed by single cell tracking.

